# Health challenges in long-distance dog sled racing: A systematic review of literature

**DOI:** 10.1080/22423982.2017.1396147

**Published:** 2017-11-02

**Authors:** Giovanna Calogiuri, Andi Weydahl

**Affiliations:** ^a^ Department of Dental Care and Public Health, Inland Norway University of Applied Sciences, Elverum, Norway; ^b^ School of Sport Sciences, UiT – The Arctic University of Norway, Alta, Norway

**Keywords:** Arctic winter sports, dogsled, mushing, ultra-endurance, wilderness medicine

## Abstract

Long-distance dog sled races, e.g. Iditarod (Alaska) and Finnmarksløpet (Northern Norway), are extremely demanding sporting events that might expose mushers, handlers and a large number of organisers and volunteers to risks for their health. The purpose of this systematic literature review (PROSPERO registration n. CRD42017069136) was to identify and summarise all available scientific literature relative to health issues connected to participating in these races. Using a literature search strategy in line with PRISMA guidelines, 117 scientific studies, sought through databases (Google Scholar and PubMed, between 2^nd^ and 9^th^ May 2017) and scrutiny of reference lists, were screened. Studies published in English treating any health issues assessed during or after a long-distance dog sled race were included, with no restriction in relation to their study design or the characteristics of participants studied. The quality of the studies was assessed using a standardised checklist. Ten studies met the criteria for being included in a qualitative analysis. The data synthesis showed that participants underwent strenuous psychophysical load, with insufficient sleep/rest and inadequate energy intake. Findings on hydrations are mixed. The risk of incurring in life-threatening injuries or infections was low, although injuries and infections of minor severity were common. No alterations of blood markers were observed from before to after the races. These findings will help planning prevention and treatment strategies in long-distance dog sled races. However, more research is needed in this field in the future.

## Introduction

Dog sled races are timed competitions of teams of sled dogs that pull a sled with a driver (*aka* musher) standing on the runners. There are different types of dog sled races, taking place on different terrains and running over different lengths. Some of these, like the Norwegian Finnmarksløp [1] and the Alaskan Iditarod [2], take place on snow and cover distances ranging from 500–1569 km in a continuous competition lasting for up to 14 days to be completed. The environmental conditions, as well as the psychophysical demands, of these races are extremely challenging: the temperature is generally below 0°C and the snow conditions can vary from icy to sugar-like. The mushers help the dogs by pulling or kicking while standing on the sled, and sometimes they might run for long distances, in particular when they are in uphill tracks. They often ride during night-time in order to move though the various check-points in the quickest way possible. Moreover, when arriving at a check-point, they have to take care of their dogs and get ready for the next leg, leaving them little time to sleep [3]. The mushers are assisted by a support team (*aka* handlers), responsible for serving the mushers and keeping time at the checkpoints during the race, coach the racers and transport equipment to and from whenever needed. The races also involve a large number of staff-members, organisers and volunteers, who have to be present on the field at the various check-points to assist the participants, control that the rules are respected, organise and facilitate the entrance and departure of the teams and in general ensure a smooth and safe development of the race. Not unlike the mushers, all the other participants (i.e. handlers, organisers and volunteers) are exposed to extreme environmental conditions and must be active and alert at all times, with brief and irregular breaks for sleeping.

**Figure 1.**
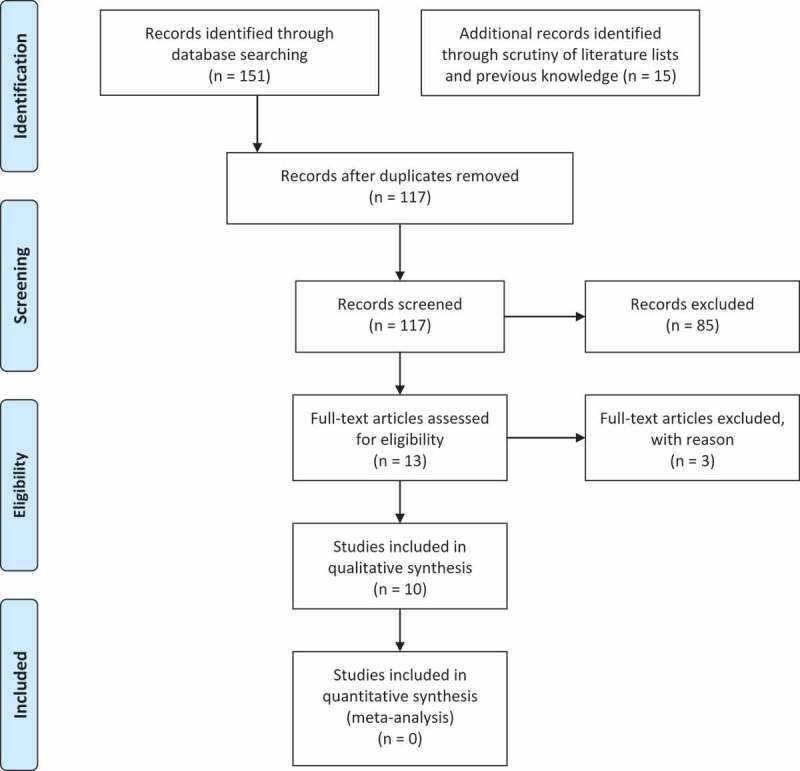
PRISMA flow diagram [8].

The extreme environmental conditions, strenuous effort and sleep deprivation imposed on mushers and the other participants may have deleterious consequences for their health. For example, it was found that participation in extreme endurance exercise of prolonged duration, such as an ultra-marathon, can lead to reduction in systolic function [4] and might adversely affect mucosal immunity and cause significant changes in the concentration of leukocyte sub-sets [5]. Participation in adventure racing (which can last up-to ~ 100 hours) can cause symptomatic injury and illness and mood state disruption, which generally resolves within a fortnight following racing [6]. It is also known that orthopaedic injuries, hypothermia, frostbite, dehydration, fatigue, acute mountain sickness and sunburn can occur during sporting activities that take place in snow environments, such as for example skiing and snow-boarding [7]. However, because of the specific characteristics and the environmental condition in which they take place, long-distance dog sled races might differ from other ultra-endurance competitions. There is, therefore, a need to identify major health hazards for these specific sporting events, in order to educate participants and organisers in how to prevent or treat them.

Thus, the overarching objective of this systematic literature review is to identify and summarise all available scientific literature on health issues among participants in long-distance dog sled races. More specifically, we aimed to: (i) identify the aspects/types of health challenges that have been investigated among participants in long-distance dog sled races and summarise up-to-date knowledge relative to such health issues; (ii) examine and evaluate common methodological approaches and challenges used by researchers in this field; and (iii) outline recommendations for participants, organisers and researchers. To the best of our knowledge, this is the first systematic review of literature on this topic. This study will provide knowledge that can help with the planning of prevention and treatment strategies in dog sled racing.

## Methods

This literature search complied with systematic review methodology in line with the PRISMA statement [8]. A protocol was registered on PROSPERO [9] (registration n. CRD42017069136). This study was entirely funded by Inland Norway University of applied Sciences and UiT – The Arctic University of Norway, as part of the authors’ research activity.

### Conceptual criteria

This systematic review focused on different health issues among participants in long-distance dog sled races. In this context, “health issue” is defined as *any physical condition or processes that might result from or that might lead to pathologies or affected states of physical or mental well-being*. The target population of this systematic review consists of all individuals directly involved in the races development; this includes mushers as well as the other participants (i.e. handlers, organisers and volunteers) present in the field during the race.

### Literature search strategy

Relevant papers were searched through digital databases and scrutiny of literature lists of selected papers. Studies that were already known by the authors were also included. A database search took place within the period 2–9 May 2017 and was conducted on PubMed and Google Scholar, which were chosen because of their pertinence with the field and their broad indexing. Key words were selected based on the authors’ knowledge of the field, as well as based on wording emerged during preliminary/exploratory searches, and included terms such as “dog sled”, “dogsled”, “musher”, “Finnmarksløp” and “Iditarod”, applying filters such as “field search for abstract/title” and considering publications only in English with an abstract. The resulting lists of references first underwent scrutiny based on title and abstract. The full-text of possible relevant papers was downloaded in digital format, if available, otherwise they were retrieved via the library system of the first author’s Institution. The search was concluded when saturation was reached (i.e. when any new attempt produced only papers that have already been selected). A detailed overview of the database searching strategy is presented in Table 1.

**Table 1.** Database search strategy.

Date	Database	Keywords	Filters	Overall results	Title/abstract screened	Full-text screened	Excluded with reason	Final included
2 May 2017	Google Scholar	Dog sled	“contains all words” “in title/abstact” patents and citations excluded	47	10	9	4 (2 no health issue investigated, 1 no empiricalevidence reported, 1 duplicate)	6
2 May 2017	Google Scholar	Finnmarkslop	“contains all words” “in title/abstract” patents and citations excluded	27	2	0	2 (duplicate)	0
2 May 2017	Google Scholar	Iditarod	“contains all words” “in title/abstract” patents and citations excluded	48	3	0	3 (duplicates)	0
7 May 2017	PubMed	“Dog sled*”	None	9	3	1	2 (duplicates)	1
7 May 2017	PubMed	Musher*	Title/abstract	11	2	0	2 (duplicates)	0
9 May 2017	PubMed	Dogsled*	Title/abstract	5	5	2	3 (duplicates)	2
9 May 2017	PubMed	Iditarod		27	4	1	3 (duplicates)	1
9 May 2017	PubMed	Finnmarksløp*		3	3	0	3 (duplicates)	0

*In Pubmed, the asterisk was used to identify key-words with different extensions.

### Selection criteria

All records were screened for eligibility and then reviewed by the first author. Only publications in English were included, with no restrictions on their study design or year of publication. Publications within the popular or popular-scientific media (e.g. magazines, internet articles, etc.) were excluded. Brief conference papers with no detailed information about methods (i.e. abstracts) and duplicative or redundant publications were also excluded. Other reasons for exclusion criteria were: (i) measurements were not conducted during the race period or in the short-term after completion of the race (i.e. within a month); (ii) the study subjects were the dogs; (iii) empirical evidence was not reported (i.e. theoretical papers); or (iv) the study did not report findings directly linked to the participants physical health (i.e. papers in the field of sport-psychology, history, economy, etc.).

### Data extraction and synthesis

Basic information were extracted from the included papers and reported on standardised spreadsheets by the first author. Major focus was given to the following aspects of each paper: (i) type of publication, (ii) study design, (iii) sample size and participants’ characteristics, (iv) health issue/s treated, (v) instruments and (vi) main findings. Major health issues treated were identified and, for each of them, separate spreadsheets were produced in order to compare the design and outcomes of the different studies and summarise the cumulative knowledge gained within each of these areas. Based on the number of studies within each health issue and the extent to which the outcomes were consistent across the studies, it was also possible to assess the strength of the body of evidence in relation to each health issue, as well as identify areas of study that received less attention. All selected papers, analysis tables and notes produced were eventually reviewed by the second author and disagreements were discussed until consensus was reached. Because the studies treated a large variety of health issues, using different instruments, we were unable to perform a quantitative synthesis of the findings; therefore, only a qualitative synthesis was performed.

### Studies quality assessment

A checklist for the studies quality assessment was developed based on eight set criteria, with a maximum total quality score of 10: (1) Study design [score: cross-sectional = 0, longitudinal = 1, descriptive field study with repeated measurements = 2]; (2) Sample size [score: 10 or less = 0; 11–20 = 1; 20 or more = 2]; (3) Inclusion of a control/comparison group [score: no = 0; yes = 1]; (4) Inclusion of baseline or pre-race assessments [score: no = 0; yes = 1]; (5) Analyses stratified or controlled for sex [score: no = 0; yes = 1]; (6) Analyses stratified or controlled for other possible confounders [score: no = 0; yes = 1]; (7) Use of valid and appropriated instruments [score: possibly biased = 0; valid and/or appropriated = 1; gold-standard = 1.5]; and (8) Self-reported or objective measurements [score: Only self-reported measurements = 0; Objective or a combination of self-reported and objective measurements = 0.5]. As most of studies investigated more than one health issue, the last two items were applied to each health issue investigated and a mean value was calculated to establish the final score. Additionally to this scoring system, a discussion of the quality of the different studies, with emphasis on the generalizability of the findings and validity of the outcome variables, was developed.

## Results

The combinations of keywords resulted in a total of 151 titles (search carried out between 2^nd^ and 9^th^ May 2017). Four additional studies co-authored by the authors of this review, which met the inclusion criteria, were also added. Eleven additional studies were identified through scrutiny of the reference lists of the included studies. After excluding for duplicates, a total of 117 references were screened based on title and abstract. Of these, 13 met the inclusion criteria and were assessed on the basis of their full text. Two papers were excluded because they did not report findings relative to the participant’s physical health and one paper was excluded because it did not report empirical findings. Thus, 10 papers were eventually included in the qualitative synthesis (Figure 1).

Of the included studies, one was a doctoral dissertation and nine were journal articles (of which two were short reports). With respect to the research design described in the different studies, eight were descriptive field studies with repeated measurements [10–17] (two of which were defined as “case” studies based on reports of one or two participants [16,17]) and two were surveys [18,19] (of which, one was designed as a perspective longitudinal study [19]). In most studies, measurements were done during or shortly before/after the race, while three studies included follow-up measurements over 1 week [10,11] and 1 month [19] after completion of the race. The studies accounted for a total of 185 subjects (accounting for overlapping samples), among which at least 66 were males and 48 were females, while for 71 subjects the sex was not specified. Most of these subjects were mushers participating in the Finnmarksløp or the Iditarod, although two studies also included other participants (i.e. handlers and race-organisers). The different studies investigated a variety of health issues, which were grouped into six categories: (i) Psychophysical load [six studies], (ii) Energy intake and hydration [six studies], (iii) Sleep and alertness [four studies], (iv) Blood markers [two studies] and (v) Injuries and infections [one study]. A summary of all included studies is presented in Table 2.

**Table 2.** Summary of papers included in the qualitative analysis.

Reference	Quality score	Race and participants	Study design	Health issue/s	Instruments	Summary of findings
Calogiuri and Weydahl [10]	5.50	10 handlers and organisers (3 M and 7 F; Age: 48±8 years), participating in the *Finnmarksløp 500K and 1000K*	Descriptive field study with baseline assessments, repeated measurements and comparisons between participants in the short and long race	Psychophysical workload Sleep & alertness	Continuous HR-monitoring Actigraphy (analysed using Activity-analysis, Sleep-analysis and NPCRA) with sleep logs Sleep questionnaire and sleepiness scale	The average estimated workload was of relatively low intensity. However, the long duration alongside the little resting time makes the race extremely demanding. During the race, the participants slept about 4-hours per day, accumulating a sleep-debt of ~ 8–28 hours. Overall, the sleep-wake patterns and alertness levels were impoverished after completion of the race, especially among the participants who were engaged in the race for a longer span.
Calogiuri et al. [11]	5.50	10 mushers (2 M and 8 F, Age: 43±11 years) participating in the *Finnmarksløp 500K and 1000K*	Descriptive field study with baseline assessments, repeated measurements and comparisons between participants in the short and long race	Psychophysical workload Sleep & alertness	Continuous HR-monitoring Actigraphy (analysed using Activity-analysis, Sleep-analysis and NPCRA) with sleep logs	The average estimated workload was of relatively low intensity. However, the long duration alongside the little resting time makes the race extremely demanding. During the race, the participants slept about 3-hours per day, accumulating a sleep debt of ~ 11–35 hours. Overall, the sleep-wake patterns were impoverished after completion of the race, especially among the participants who were engaged in the race for a longer span, who also showed an impoverishment of sleep quality.
Calogiuri et al. [19]	5.00	55 handlers and organisers (38 M and 17 F, Age: 44±13 years) participating in the *Finnmarksløp 500K and 1000K*	Longitudinal study, with comparisons between participants in the short and long race	Sleep & alertness	Online survey including a PSQI and additional items	During the race, most participants slept 4-hours or less per day, accumulating a sleep debt of ~ 11–35 hours. Despite most participants being aware of having accumulated a sleep debt, many did not engage in adequate strategies to recover the sleep loss. “Keeping regular sleep-wake routines” was a common strategy to recover from sleep problems, while “going to bed earlier” and “sleeping whenever possible” and “taking naps during the day” were common sleep-augmentations strategies.
Case et al. [12]	4.50	10 mushers (7 M and 3 F, Age: n.r.) participating in the *Iditarod*^a^	Descriptive field study with baseline assessments and repeated measurements	Energy intake and hydration Blood markers	BIA and body weight assessment Urine specific gravity Serum concentration of Thyroid hormones (only in 5 mushers)	There was a decrease in body weight and body fat percentage and an increase in total body water from pre- to post-race. No significant change in lean body mass or fluid balance was detected. The profile of the thyroid hormones did not change significantly, indicating that the strenuous activity mitigated against the classic perturbation in thyroid axis brought on by the hypocaloric state.
Chapman et al. [13]^a^	6.50	16 mushers (13 M and 4 F, Age: 26–53 years) participating in the *Iditarod*	Descriptive field study with baseline assessments and repeated measurements	Energy intake and hydration	BIA and body weight assessment	There was an average body fat loss in both male and female racers. Total body water and lean body mass remained stable, while body water percentage increased.
Cox [14] Chapter 3	3.00	5 mushers (1 M and 4 F, Age: n.r.) participating in the *Iditarod*^a^	Descriptive field study with repeated measurements	Psychophysical workload Energy intake and hydration	Doubly labelled water Self-reported food intake recording	The mushers had a total energy expenditure above that estimated for the normal population and comparable to wildland firefighters, Marines during field exercise or soldiers during arctic exercise. The energy expenditure increased during the last leg of the race, likely due to fatigued dogs. Food records indicate an adequate intake of protein, but an insufficient intake of carbohydrate.
Cox et al. [15]	5.67	16 mushers (9 M and 7 F, Age: 36±11 years) participating in the *Iditarod*^a^	Descriptive field study with baseline assessments and repeated measurements	Psychophysical workload Sleep & alertness Energy intake and hydration	RPE, Fatigue scale, resting HR and HR response to a bench step exercise test COWA Sampling or urine, analysed for specific gravity, osmolality and isotopic analysis Body-weight assessment (digital scale)	Resting HR (but no HR response), RPE and fatigue increased throughout the course of the race and some associations were observed between these parameters and the mushers’ hydration status. Most mushers were dehydrated at some point of the race. The average water turnover was similar to that of cyclists, but lower compared to other activities such as trekking, climbing and wildland fire suppression. The mushers’ body weight tended to reduce from pre- to post-race, although with some inter-individual differences.
Cox et al. [16]	5.67	One female musher (Age: 49 years) participating in the *Iditarod*	Case study with baseline assessments and repeated measurements	Psychophysical workload Energy intake and hydration Blood markers	Simplified food journal Haematocrit and haemoglobin profile Skinfold measurements, hydrostatic weighing and simple weight assessment	The energy demands of the race were high. The carbohydrate intake of the musher was low (as opposed to what would be recommended for sporting activity of such levels of endurance). Signs of dehydration were observed, mainly noticed by a rapid increase in body-weight within the 24-hours following completion of the race.
Gallea et al. [18]	3.00	71 mushers (sex and age: n.r.) in the *Iditarod*.	Cross-sectional study	Injuries and infections	Survey	Most injuries and illnesses were minor and self-treatable. Injuries were more frequent than infections. The majority of respondents reported some injury, most commonly frostbite and Musculoskeletal pain. Acute upper respiratory infections were also somewhat frequent. In most instances, medical conditions were self-treated, otherwise care was administered by race veterinarians, support staff and local village clinicians.
Weydahl and Calogiuri [17]	5.50	Two female mushers (Age: 23–53 years) participating in the *Finnmarksløp 1044K*	Case study with repeated measurements	Psychophysical workload Energy intake and hydration	Continuous HR-monitoring and RPE assessment Sampling or urine, analysed for specific gravity BIA, skinfold measurements, body weight and anthropometric assessments Drink and food log	The workload was, on average, of moderate intensity, although both participants reached at times very high levels of intensity. The amount of liquids consumed was adequate to supplement hydration losses, but one of the mushers was in a poor hydration state from *before* entering the race. This participant showed increasing RPE throughout the race and eventually withdrew half-way. Only small changes of the anthropometric measurements were found from pre- to post-race.

^a^ Studies with overlapping samples.

BIA = Bioelectric impedance analysis; COWA = Controlled oral word association; HR = Heart rate; NPCRA = Non-parametric circadian rhythm analysis; PSQI = Pittsburgh sleep quality index; RPE = Ratings of perceived exertion.

### Health issues in participants in long-distance dog sled races

#### Psychophysical load

In sports science and practice, *load* is defined as “the sport and non-sport burden (single or multiple physiological, psychological or mechanical stressors) as a stimulus that is applied to a human biological system […] over a varying time period” [20]. The concept of load plays an important role in relation to performance-enhancing training programmes, but also in relation to prevention of injuries and illness: higher load can expose athletes to increased risk of injury and illness, but on the other hand a rational pacification of the training in order to prepare the athlete to face the load requirements of a competition can provide a protective effect [21,22]. Evidence about the physical and cognitive demands of long-distance dog sled races can, therefore, help participants to prepare adequately in view of the event, reducing the risk of incurring injuries or illnesses.

Three studies by Calogiuri and Weydahl have carried out continuous monitoring of the participants heart rate (HR) throughout the race (or part of it) and cross-matched this information with the official race reports in order to estimate the exercise intensity during the different legs [10,11,17]. These studies reveal that, on average, the exercise intensity sustained by the participants in long-distance dog sled races is not particularly high *per se*: the mean HR measured during the legs ranges between 50% and 65% of the participants maximal HR, although at times very high levels of intensity were achieved (HR above 90% of the maximum) [10,11,17]. However, these studies also reveal that the participants engage in few and brief bouts of rest (on average two bouts per day, accounting for an overall rest-time of about 3–4 hours per day) [10,11], which, alongside the long duration of the race and the challenging environmental conditions, makes the overall load quite strenuous. These findings are in line with two studies carried out by Cox and her colleagues, in which the energy expenditure of the participants was assessed using doubly labelled water [14] and by combining energy intake data with weight loss data [16]. These studies demonstrate that the overall energy demands required by these long-lasting races are high, and comparable to that of wildland firefighters, Marines during field exercise, or soldiers during arctic exercise [14,16]. The energy expenditure of the mushers especially increased during the last leg of the race, likely due to fatigued dogs and/or a reduction of the dogs pack, which put more strain on the mushers [14]. However, findings on perceived exertion show that the subjective experience of fatigue can vary among the participants, and indicate that such subjective experience of exertion/fatigue might be associated with physiological parameters such as the participants’ hydration status [15,17].

One study found some differences were observed between participants in shorter or longer races (i.e. Finnmarksløp 500K and 1000K, respectively): the mushers in the shorter race engage in higher exercise intensities as compared to the mushers in the longer race [11]. Such differences in load probably depend on the fact that the mushers in the shorter race are allowed a smaller number of dogs than the musher in the longer race (seven and 12, respectively), but it could also be explained by different pacing strategies adopted by the participants in the different races. Studies show in fact that athletes tend to distribute their effort in a purposeful fashion, knowing the duration of a given exercise [23].

Most of the studies investigated psychophysical load in the mushers [11,14–17], while only one study [10] studied this health issue in handlers and race-organisers, revealing that this group of participants is also exposed to quite demanding volumes of load. For example, while handlers and race-organisers are not directly involved in mushing activities, their exercise intensity (HR ranged around 50% of their HR maximum [10]) do not differ greatly from that found in other studies among mushers [10,11,17]. Moreover, not unlike the mushers, these participants engage in little rest: about 4 hours per day (usually divided in two bouts per day) during the entire course of the race [10]. On the other hand, unlike the mushers, the exercise intensity did not differ between those involved in the shorter and longer race [10].

#### Energy intake and hydration

Sports medicine institutions recommend consuming adequate amounts of liquids and macronutrients during exercise in order to prevent excessive dehydration and changes in electrolyte balance that might compromise performance as well as the organism’s well-functioning [24]. Because of the continuous engagement and long duration nature of these competitions, participants in long-distance dog sled races might be particularly exposed to risks of dehydration and insufficient intake of macro- and micro-nutrients. Moreover, access to drinks and food can be difficult, as the participants wear several layers of insulating clothing, store liquid in containers and have to balance on the sled.

Overall, the studies consistently indicate that the mushers tend to have an insufficient energy intake during the race, especially in terms of the amount of carbohydrates consumed [14,16]. This is associated with a loss of body fat from pre- to post-race, although studies show that lean body mass is usually maintained [12,13,16,17]. On the other hand, the findings regarding the ability of the mushers to consume sufficient amounts of liquids in order to maintain optimal levels of hydration are mixed. For example, Chapman *et al*. [13] and Case *et al*. [12] found that total body water was maintained from pre- to post-race. In contrast, in a case study, Cox *et al*. [16] observed signs of dehydration, with liquid loss being quickly restored within 24-hours after completion of the race. It is, however, likely that drinking behaviour and consequent hydration status varies, largely depending on individuals and situations. For example, Cox *et al*. [15] found that, while on average their sample of mushers maintained optimal levels of hydration, most of them (77%) showed some sign of dehydration at some point during the race. Weydahl and Calogiuri [17] also observed that two female mushers assumed adequate amounts of liquids throughout the race, but one of them was in a state of dehydration from before the beginning of the race.

Only one study investigated possible differences between male and female mushers in the extent to which they consume adequate amounts of nutrients and liquids during the race. Chapman et al. [13] found that male mushers were subjected to a larger loss of body fat compared with female mushers [13], although this finding could have been influenced by pre-existing differences. Weydahl and Calogiuri [17] also suggested, based on a comparison of their findings with previous literature, that female mushers might tend to consume more liquid than male mushers, although evidence supporting such an assumption is missing. Cox *et al*. [15] and Weydahl and Calogiuri [17] also speculate that less experienced mushers are more exposed to the risks of dehydration, as they haven’t established good routines for taking on adequate amounts of liquids before and during the race.

#### Sleep and alertness

Sleep loss and disrupted alertness can represent a hazard for participants in long-distance dog sled races, exposing them to an increased risk of accidents. Studies have in fact consistently shown that sleep loss, resulting for example from shift-working schedules, is associated with increased risk of accidents and, in the long-term, even health consequences such as type-2 diabetes and cancer [25]. Furthermore, when recurrent sleep loss is not followed by adequate sleep recovery, it can lead to increased risk of infections as well as chronic conditions [26]. In a series of studies, Calogiuri and Weydahl have estimated that participants in long-distance dog sledding competitions sleep, on average, 3–4 hours per day, usually divided into two separate bouts during the day [10,11,19]. They calculated that mushers in the 1000 km Finnmarksløp accumulate, in general, a sleep debt grossly equivalent to 35-hours over a 6-day span, whereas other participants (handlers and race-organisers) can accumulate a sleep debt grossly equivalent to 28-hours in the same period.

Cox *et al*. [15] hypothesised that such a tight schedule would have a negative impact on the mushers cognitive functions, which could, in turn, lead to a reduced ability to engage in safety procedures, such as assuming adequate amounts of liquids and nutrients. In their study, they administered a cognitive test (the Controlled oral word association) to a sample of mushers at different check-points throughout the race, but failed, however, in finding evidence for such relationship [15]. On the other hand, Calogiuri and Weydahl have shown that the sleep loss accumulated during the race has long-distance effects on the participants’ alertness and sleep-wake patterns [10,11,19]. They found, for example, that the perceived sleepiness at bed time was increased for a week after completion of the race [10] and that most participants reported having trouble staying awake and that “Lack of energy” was a problem for up to 1 month after completion of the race [19]. Recovering from such a condition was hindered by the fact that many participants did not engage in appropriated sleep-recovery strategies, which was likely partly due to a disruption of their physiological sleep-wake rhythms [10,11], but also partly because of a lack of awareness of having accumulated a sleep debt or knowledge on how to make it up [19]. The severity of the sleep loss consequence was greater in participants who were engaged in the race for a longer span than those who were engaged in the race for a shorter span [10,11,19].

#### Blood markers

Examination of blood markers is a common procedure for testing individuals’ health status. However, the findings of our systematic review show that only two studies have investigated possible effects of undergoing long-distance dog sledding races on the participants’ blood markers. In a case study, Cox *et al*. [16] measured haematocrit and haemoglobin levels in a female musher before and after participating in the Iditarod. These blood markers were maintained within normative values from pre- to post-race [16]. Case *et al*. [12] measured the serum concentration of hormones (total and free thyroxine and triiodothyronine – TT4, TF4, TT3 and FT3, respectively) in five mushers before and after participating in the Iditarod, hypothesising that the insufficient energy intake to which the mushers were exposed would have affected the profile of these hormones. The study found, however, no significant changes in these hormones, speculating that the strenuous activity might have mitigated the expected perturbation in thyroid axis brought on by the hypocaloric state [12].

#### Injuries and infections

Sport-related injuries are common and often include sprains and strains, knee injuries, swollen muscles, Achilles tendon injuries, pain along the shin bone, fractures and dislocations. Some are caused by accidents, but injuries may also result from poor practices, improper gear or unfavourable environmental conditions. People may be especially at risk of injury in cold environments, although with adequate precautions such types of injuries might be prevented [27]. Engaging in strenuous exercise while being exposed to cold temperatures might also elicit the development of infections, such as infections of the respiratory tracts. Gallea *et al*. [18] carried out a survey among participants in the Iditarod in order to assess the incidence of injuries and infections among the mushers. They found that most injuries and illnesses sustained by mushers in the Iditarod are minor and self-treatable, most commonly frostbite and musculoskeletal pain, with life-threatening conditions being rare. Moreover, despite the poor sanitary conditions, infections, which most commonly consisted of upper respiratory infections, were also relatively infrequent. In most instances, medical care (such as wound care and administration of oral antibiotics) was self-administered or provided by race veterinarians, support staff and clinicians in local villages. Based on these findings, the authors concludes that the need for an organised medical care system in this type of event seems low.

## Quality of the studies

The total quality score is presented in Table 2. The quality score ranged between 3 and 6.50, indicating a generally low quality of the studies. Most studies were especially weak with respect to sample size, inclusion of a control or comparison group and controlling for possible confounders such as sex and age.

### Generalizability of the findings

All studies selected were field studies, with descriptive or quasi-experimental designs. None of the studies included a control group, although, in three studies [10,11,19], the authors made some comparisons between different categories of participants, i.e. those who were engaged in the race for a shorter or a longer span, in an attempt to emphasise possible dose-response effects. In the studies, the authors fail, however, to control for possible confounders such as sex and age, which might have influenced the findings. For example, the participants in shorter races were on average younger than the participants in the longer races and this might have acted as a confounder, as sleep-wake patterns can change in different age groups. In most studies, and especially in the two case studies [16,17], the sample sizes were small and not well balanced with respect to sex (males/females). Two surveys [18,19] managed to enrol larger samples, although it appears they might have been subjected to response bias: in both studies, the surveys were administered at two different time points, with some participants responding at both time-points, while other responded only at one of the two time-points. Self-reported measurements might also be subjected to recall bias, especially considering that the participants were in a state of exhaustion and dehydration. It should, however, be considered that research in this field meets a number of challenges, not least ethical ones: attempting to simulate the conditions met by the participants in long-distance dog sledding races in more controlled settings (e.g. race-simulations) would meet a number of barriers in term of organisational and ethical barriers. On the other hand, the studies examined in this review, especially those in which repeated measurements were collected before, during and after the race in groups of mushers [10–12,14,15], provide at least a quite good degree of *ecological* validity.

### Validity of the outcome variables

The studies selected relied on a variety of self-reported (e.g. food logs, sleep logs and questionnaires) and “objective” measurements (e.g. urine and blood samples and anthropometric assessments) and this can be viewed as a strength. In fact, while self-reported measurements are often considered limited in their validity because of possible recall or response bias, they can provide greater understanding of the subjective experiences of the participants [28]. The combination of self-reported and objective measurements is especially useful when investigating the participants’ load, food and liquids intake and sleep/alertness states. The quality and validity of the instruments varied, however, among the studies. For instance, physical load was assessed using heart rate monitoring [10,11,17], energy intake based on self-reported food logs [16], and doubly-labelled water [14], the latter being considered as having great validity for use in field studies [29]. Body mass composition has been assessed using skinfold measurements [16,17] or bioelectric impedance analysis [12,13,17], the latter being considered as having greater validity compared with the former, although this might vary depending on the device and type of equation used [30]. For example, Weydahl and Calogiuri [17] used a hand-to-hand device, which is considered as having smaller validity as compared with the full-body assessments used by Case *et al*. [12] and Chapman *et al*. [13].

## Overall discussions

The 10 studies selected in this systematic literature review investigated a variety of health issues, categorised into five major groups: (i) Psychophysical load, (ii) Energy intake and hydration, (iii) Sleep and alertness, (iv) Blood markers and (v) Injuries and infections. The studies included relatively small samples and used a variety of instruments with different levels of validity. They all adopted descriptive or field designs, most of which included repeated assessments before, during and after the race. No study included a control sample, although some made comparisons between different categories of participants, in order to establish possible dose-response effects due to the duration of the race. The studies are, therefore, deemed to have a limited generalizability, although they show a fairly good *ecological* validity.

Cumulative knowledge in this field remains limited, especially concerning the health issues relative to blood markers and incidence of injuries and infections, while there appears to be adequate evidence in relation to psychophysical load and energy intake. Mixed findings have been found in relation to the participant’s hydration state and drinking behaviour, especially in relation to possible correlates of or influential factor. Relatively abundant evidence is provided in relation to sleep loss during the race and post-rase sleep recovery, but virtually no evidence is available in relation to the participants’ alertness state during the race and whether such states are associated with other health outcomes such as drinking/eating behaviours, development of infections and occurrence of injuries. Moreover, no study has investigated health issues such as inflammatory markers or cardiovascular parameters, which have been found to be altered in ultra-marathon participants [4,5]. This is in spite of the fact that the need to investigate possible cardiac functions among mushers has been advocated previously [31]. There is also very little evidence of possible differences between male and female participants, as well as possible health problems among other participants assisting in the race (handlers, organisers and volunteers).

## Conclusions

Long-lasing dog sled races are extremely demanding and expose the participants to strenuous physical activity, sleep loss, inadequate energy intake and high risk of incurring injuries or infections, although the latter are usually of minor severity. Findings about the participant’s hydration state are mixed, while there is little evidence concerning possible effects on different blood markers. In general, knowledge about possible health issues in this population is still limited. It is especially recommended that future research investigates possible effects of competing in long-distance dog sled races on cardiac functions and inflammatory markers. Studies should also seek to control for possible confounding effects due to sex, age and racing experience.
